# Tissue lipidomic profiling supports a mechanistic role of the prostaglandin E2 pathway for albuminuria development in glomerular hyperfiltration

**DOI:** 10.3389/fnetp.2023.1271042

**Published:** 2023-12-22

**Authors:** Debora Kaiser-Graf, Angela Schulz, Eva Mangelsen, Michael Rothe, Juliane Bolbrinker, Reinhold Kreutz

**Affiliations:** ^1^ Corporate Member of Freie Universität Berlin and Humboldt-Universität zu Berlin, Institute of Clinical Pharmacology and Toxicology, Charité—Universitätsmedizin Berlin, Berlin, Germany; ^2^ Lipidomix GmbH, Berlin, Germany

**Keywords:** albuminuria, glomerular filtration, glomerular hyperfiltration, chronic kidney disease, lipidomic profiling, prostaglandin E2 pathway, Munich Wistar Frömter rats

## Abstract

**Background:** Glomerular hyperfiltration (GH) is an important mechanism in the development of albuminuria in hypertension. The Munich Wistar Frömter (MWF) rat is a non-diabetic model of chronic kidney disease (CKD) with GH due to inherited low nephron number resulting in spontaneous albuminuria and podocyte injury. In MWF rats, we identified prostaglandin (PG) E_2_ (PGE_2_) signaling as a potential causative mechanism of albuminuria in GH.

**Method:** For evaluation of the renal PGE_2_ metabolic pathway, time-course lipidomic analysis of PGE_2_ and its downstream metabolites 15-keto-PGE_2_ and 13-14-dihydro-15-keto-PGE_2_ was conducted in urine, plasma and kidney tissues of MWF rats and albuminuria-resistant spontaneously hypertensive rats (SHR) by liquid chromatography electrospray ionization tandem mass spectrometry (LC/ESI-MS/MS).

**Results:** Lipidomic analysis revealed no dysregulation of plasma PGs over the time course of albuminuria development, while glomerular levels of PGE_2_ and 15-keto-PGE_2_ were significantly elevated in MWF compared to albuminuria-resistant SHR. Overall, averaged PGE_2_ levels in glomeruli were up to ×150 higher than the corresponding 15-keto-PGE_2_ levels. Glomerular metabolic ratios of 15-hydroxyprostaglandin dehydrogenase (15-PGDH) were significantly lower, while metabolic ratios of prostaglandin reductases (PTGRs) were significantly higher in MWF rats with manifested albuminuria compared to SHR, respectively.

**Conclusion:** Our data reveal glomerular dysregulation of the PGE_2_ metabolism in the development of albuminuria in GH, resulting at least partly from reduced PGE_2_ degradation. This study provides first insights into dynamic changes of the PGE_2_ pathway that support a role of glomerular PGE_2_ metabolism and signaling for early albuminuria manifestation in GH.

## 1 Introduction

**FIGURE 1:**
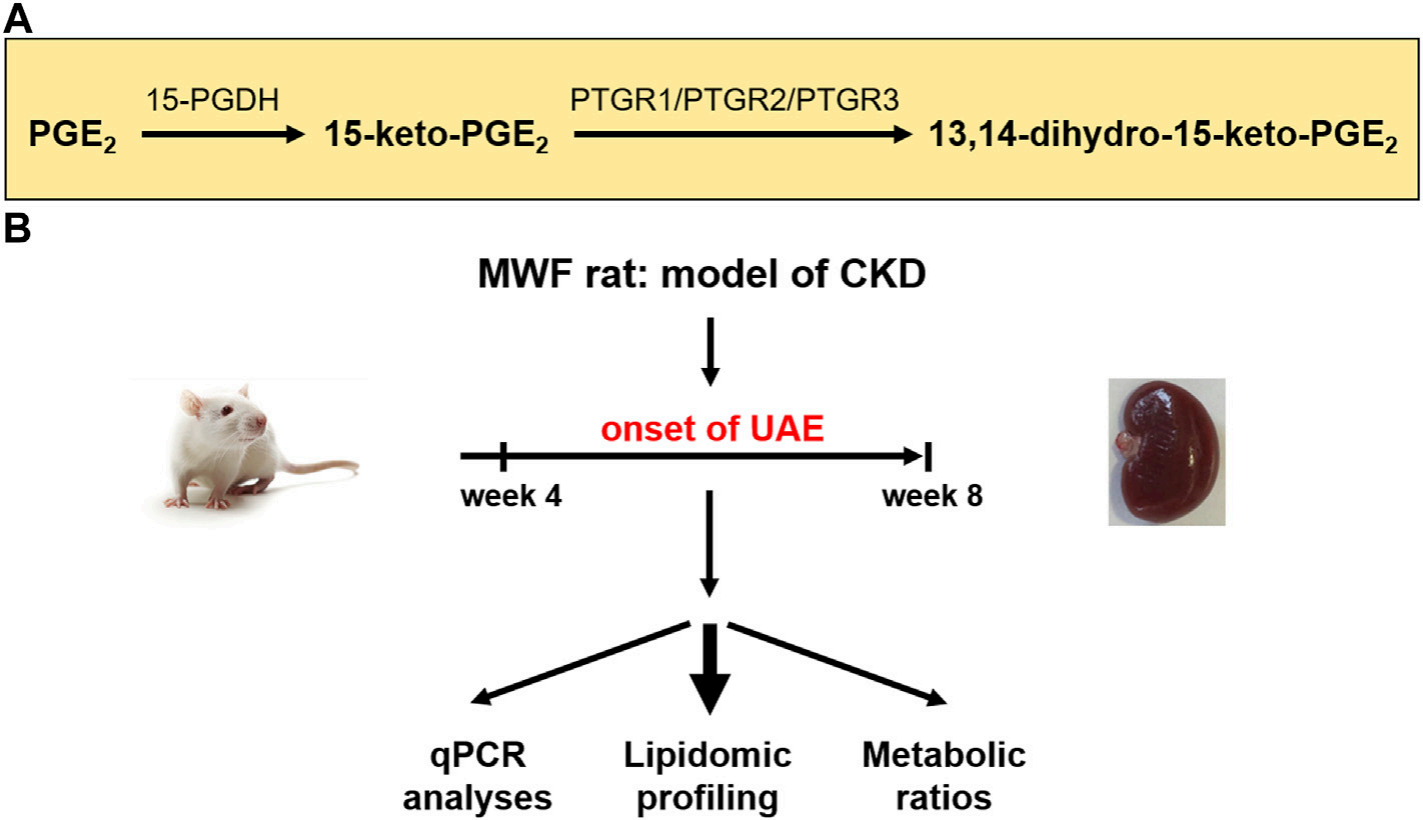
Study design for evaluation of prostaglandin metabolism in the chronic kidney disease (CKD) rat model Munich Wistar Frömter (MWF). **(A)** Schematic representation of enzymatic reduction of both prostaglandin E2 (PGE_2_) and 15-keto-PGE_2_ by prostaglandin dehydrogenase (15-PGDH) and prostaglandin reductases 1–3 (PTGR1-3) (Wu et al., 2008). **(B)** Young male MWF rats were studied between 4 weeks and 8 weeks of age and were compared to the reference strain spontaneously hypertensive rats (SHR). Therefore, lipidomic profiling and transcriptomic analysis of the PGE_2_ metabolic pathway were conducted. UAE = urinary albumin excretion.

Albuminuria is a leading symptom of the progression of chronic kidney disease (CKD) and early kidney injury (Srivastava et al., 2010; Carrero et al., 2017). Elevated fluid flow shear stress (FFSS) in Bowman’s space causes podocyte damage during glomerular hyperfiltration (GH) (Sharma et al., 2017). Prostaglandins (PGs) and particularly prostaglandin E2 (PGE_2_) play various complex roles in the manifestation and progression of CKD (Nasrallah et al., 2014; Sharma et al., 2017). In this context, COX2 (cyclooxygenase 2) and PGE_2_ activation contributes to impairment of the glomerular filtration barrier (GFB), i.e., foot process effacement of podocytes and slit diaphragm damage in albuminuria development (Srivastava et al., 2014). Furthermore, PGE_2_ signaling via the prostaglandin receptors (EP) 2 and EP4 is connected to podocyte responses during FFSS, and to kidney injury in the solitary kidney (Srivastava et al., 2014). Recently, we demonstrated a potential association between glomerular PGE_2_ accumulation and albuminuria development due to podocyte damage (Mangelsen et al., 2020). Thus, PGE_2_ degrading enzymes may play a critical role in the autocrine/paracrine COX2/PGE_2_ pathway in the development of albuminuria. Arachidonic acid is converted into PGE_2_ by cyclooxygenase enzymes 1 and 2 (COX1/COX2) and prostaglandin E synthases (PGES) (Smith, 1989; Park et al., 2006). These enzymes are expressed in all main cell types of the glomeruli (Sraer et al., 1979; Petrulis et al., 1981; Hirose et al., 1998; Harding et al., 2006; Xu et al., 2006; Yu et al., 2016; Ma et al., 2019; Yao et al., 2019). PGE_2_ is metabolized by 15-prostaglandin dehydrogenase (15-PGDH) to 15-keto-PGE_2_. The latter is terminally degraded by prostaglandin reductases PTGR1, PTGR2, and PTGR3 to 13,14-dihydro-15-keto-PGE_2_ (Figure 1A) (Tai et al., 2002; Wu et al., 2008; Yu et al., 2013; Chen et al., 2018).

To further investigate the renal PGE_2_ metabolic pathway we used the Munich Wistar Frömter (MWF) inbred rat strain as a suitable model for GH (Ulu et al., 2009; Schulz and Kreutz, 2012), which mirrors several phenotypes observed in patients with albuminuria and CKD. The glomerular hyperfiltration phenotype of MWF rats is due to an inherited nephron deficit with increased single nephron glomerular filtration rate and hemodynamically associated with dilatation of the afferent arteriole and normal glomerular capillary pressures (Remuzzi et al., 1988; Fassi et al., 1998) Overall glomerular filtration in young rats, as studied in this report is similar to normal rat strains (Rovira-Halbach et al., 1986; Fassi et al., 1998; van Es et al., 2011) MWF rats develop moderate hypertension, spontaneous albuminuria of early onset, and a congenital nephron deficit (Schulz and Kreutz, 2012). Podocyte damage with focal and segmental foot process effacement and a reduced podocyte number were early found in young adult MWF animals (Ijpelaar et al., 2008; Kourpa et al., 2023). With increasing age further significant structural renal abnormalities such as glomerulosclerosis and renal interstitial fibrosis with progressive albuminuria development were observed (Schulz and Kreutz, 2012).

So far, elevated plasma levels of circulating PGE_2_ were mostly measured by enzyme-linked immunosorbent assays (ELISA) and have been implicated in the MWF rat models of GH (Ulu et al., 2009). ELISA are often used despite their limitations including low specificity and selectivity as well as a lack of standardization across different ELISA kits (Faupel-Badger et al., 2010; Gandhi et al., 2017). Therefore, we previously developed a modified liquid chromatography electrospray ionization tandem mass spectrometry (LC/ESI-MS/MS) protocol that allows more precise quantification of PG levels (Mangelsen et al., 2020). Preliminary measurements in plasma and glomeruli of MWF rats at the onset of albuminuria suggest an implication of the PGE_2_ pathway for albuminuria development (Mangelsen et al., 2020). This supports the need to conduct comparative analysis of PGE_2_ and its downstream metabolites in more detail with state-of-the-art LC/ESI-MS/MS methodology in different kidney tissues and body fluids in the time window of albuminuria development in MWF rats (Figure 1B).

In this study, we aim to elucidate the PGE_2_ pathway in plasma, urine and glomerular tissue to clarify the impact of PGE_2_ metabolism in the context of GH during the onset of albuminuria.

## 2 Methods

### 2.1 Animals and phenotyping

Male MWF rats were obtained from our MWF/Rkb (RRID:RGD_724569, laboratory code Rkb, http://dels.nas.edu/ilar/). Male rats were used because a sexual dimorphism is conspicuous in the MWF strain with a more severe manifestation and progression of albuminuria and subsequent renal failure compared with females (Schulz and Kreutz, 2012; Herlan et al., 2015).

As a contrasting model we used male spontaneously hypertensive rats (SHR) from our SHR/Rkb (RRID:RGD_631696, laboratory code Rkb, http://dels.nas.edu/ilar/) colonies at the Charité—Universitätsmedizin Berlin, Germany (Schulz and Kreutz, 2012). SHR rats exhibit similar blood pressure values in early life (Schulz et al., 2003), but an albuminuria-resistant phenotype compared to MWF rats (Schulz and Kreutz, 2012).

Rats were grouped under conditions of regular 12 h diurnal cycles with an automated light switching device and climate-controlled conditions at a room temperature of 22°C. The rats were fed a normal diet containing 0.2% NaCl and had free access to food and water.

Urinary albumin excretion (UAE) was determined in metabolic cages for 24 h at 4 and 8 weeks of age as reported (*n =* 10–18, each) (Kreutz et al., 2000) and urinary creatinine levels (Supplementary Figure S1) were determined by the Jaffé method (Labor Berlin–Charité Vivantes GmbH, Berlin, Germany). Systolic blood pressure (SBP) was determined by a tail-cuff method in awake animals at 8 weeks of age (*n* = 5–8, each) using a computer-assisted oscillatory detection device (TSE, Bad Homburg, Germany) (Kreutz et al., 2000). Animals were sacrificed under ketamine-xylazine anesthesia (87 and 13 mg/kg body weight, respectively). For quantitative real-time PCR analysis (qPCR) and lipidomic profiling by LC/ESI-MS/MS (Mangelsen et al., 2020) cortex dissections of left kidneys, isolated glomeruli, plasma, and urine of MWF and SHR at both 4 and 8 weeks of age were snap-frozen and stored at −80°C.

### 2.2 Ethics approval statement

All experimental work in rats was performed in accordance with the guidelines of the Charité—Universitätsmedizin Berlin and the local authority for animal protection (Landesamt für Gesundheit und Soziales, Berlin, Germany) for the use of laboratory animals. The registration numbers for the rat experiments are G 0255/09, G 0309/19, G 0130/16, and T 0189/02.

### 2.3 Glomeruli and kidney cortex isolation

Different protocols were used for glomeruli isolation from rats at 4 and 8 weeks of age, due to the different body size (Schulz et al., 2019). Rats were anesthetized with ketamine-xylazine (87 and 13 mg/kg body weight, respectively). In 4-week-old rats, the abdominal artery was catheterized and kidneys were perfused with 10 mL 1x phosphate buffered saline (PBS) and subsequently with 20 mL ferrous solution 12.5 g ferric oxide [Iron (II/III) powder <5 micron, 98%; Sigma- Aldrich Chemie GmbH] suspended in 1,000 mL 1x PBS. Kidneys were removed and decapsulated. For cortical analysis the cortex of the left kidney was dissected and immediately snap-frozen and stored at 80°C. For glomeruli isolation decapsulated kidneys were passed through a 125 µm steel sieve (Retsch GmbH) with 1x PBS. The glomeruli containing ferrous particles were gathered by a magnet, snap-frozen and stored at −80°C. Kidneys of 8-week-old rats were removed, decapsulated and passed through a 125 µm steel sieve with 1x PBS. The filtrate was put on a 71 µm steel sieve (Retsch GmbH) to separate glomeruli from the flow-through. Glomeruli were washed off the sieve with 1x PBS, centrifuged, immediately snap-frozen and stored at −80°C.

### 2.4 Reverse transcription and qPCR

Isolated glomeruli preparations of rat strains were analyzed at week 4 and week 8 (*n* = 5–11, each). Total RNA of glomeruli preparations was isolated and DNase-treated by RNeasy Purification Kits (Qiagen), according to the manufacturer’s instructions. First-strand cDNA synthesis was carried out on 2 µg of total RNA using the First Strand cDNA Synthesis Kit (Fermentas Life Sciences) following the manufacturer’s protocol. qPCR analysis of mRNA expression of PGE_2_ degrading enzymes *15-Pgdh, Ptgr1*, *Ptgr2*, and *Ptgr3* was performed using the comparative quantification cycle method as reported (Schulz et al., 2019). In addition, different glomerular compartments in isolated glomeruli were evaluated by qPCR. We assessed alpha actinin 4 (*Actn4*) as a podocyte marker, platelet derived growth factor receptor beta (*Pdgfrb*) as a mesangial marker, and platelet endothelial cell adhesion molecule 1 (*Pecam1, CD31*) as an endothelial marker (Supplementary Figure S2). The extent of contamination with tubular material in isolated glomeruli was assessed by evaluating the tubular marker solute carrier family 5 member 1 (*Slc5a1, Sglt1*) (Supplementary Figure S2). Primers are listed in Table 1. Normalization of expression data was done by the reference gene hydroxymethylbilane synthase (*Hmbs*, *Pbgd*) (Schulz et al., 2008). All analyses were performed in three technical replicates for each animal or experiment (*n* = 5, each).

**TABLE 1:** Rat primer list for quantitative real-time PCR analysis.

Gene	Ensembl no.	Sense primer	Antisense primer	Amplicon (bp)	Exon
*Actn4*	ENSRNOG00000020433	TAC​GAC​GTG​GAG​AAT​GAC​CG	GAA​GGC​TTG​GAA​GGT​CAC​GA	99	19 + 20
*Pdgfrb*	ENSRNOG00000018461	GAA​GCA​GCC​ATG​AAC​CAG​GA	GTC​CTC​AGA​GTC​CAT​CGG​GA	189	3 + 4
*Pecam1*	ENSRNOG00000066008	GAA​TTC​CCC​ATC​GAG​GAG​CA	TGG​AAA​TTC​CTG​GGC​CAA​GT	231	4 + 5
*Ptgr1*	ENSRNOT00000020335	TGG​GAA​TGG​ACT​GAG​AAA​GC	GTC​AAG​CAG​GCC​AAA​GTA​GG	112	5 + 6
*Ptgr2*	ENSRNOT00000058095	GGA​GTG​GAT​GTT​TAC​TTT​GAC​AAT​G	GTC​TCC​TTG​ATC​TGA​CTT​ATC​A	71	6 + 7
*Ptgr3*	ENSRNOT00000021729	CCG​GAA​CCG​ATT​CGT​TGG​TA	GCA​CCA​CTG​TAT​ACT​CGG​CA	206	1 + 2
*Slc5a1*	ENSRNOG00000017775	CCG​TCT​GTG​CTG​GAG​TCT​AC	CCT​TTA​TCC​TGG​TCC​AGC​CC	183	14 + 15
*15-Pgdh*	ENSRNOT00000014229	AGC​GGT​GTG​AGA​CTG​AAT​GT	CAT​TGG​CAA​TGG​CTG​ATG​GG	163	6 + 7

*Actn4*, Actinin alpha 4; *Pdgfrb*, platelet derived growth factor receptor beta; *Pecam1 (CD31)*, platelet and endothelial cell adhesion molecule 1; *Ptgr1-3*, prostaglandin reductases 1–3; *Slc5a1 (Sglt1)*, solute carrier family 5 member 1; *15-Pgdh,* 15-prostaglandin dehydrogenase; bp, base pairs. Primers were designed by Primer3plus (Untergasser et al., 2012).

### 2.5 LC/ESI-MS/MS for prostaglandin profiling

#### 2.5.1 Sample preparation

Sample preparation was described previously in detail (Mangelsen et al., 2020). Briefly, for lipidomic analyses 100 µL rat urine (*n =* 8–10 animals per strain, each) were spiked with an internal standard. 500 μL methanol and 5 µL 2,6-Di-tert-butyl-4-methylphenol (BHT 10 mg/mL) was added and shaked vigorously. The samples were brought to pH 6. After centrifugation, the obtained supernatant was added to the Bond Elute Certify II columns (Agilent Technologies) for Solid phase Extraction SPE, which were preconditioned with 3 mL methanol, followed by 3 mL of 0.1 mol/L phosphate buffer containing 5% methanol (pH 6). The SPE-columns were then washed with 3 mL methanol/H_2_O (40/50, vol/vol). For elution 2 mL of n-hexane: ethyl acetate 25:75 with 1% acetic acid was used. The eluate was evaporated on a heating block at 40 °C under a stream of nitrogen to obtain solids, which were dissolved in 100 µL methanol/water 60:40 and transferred in an HPLC autosampler vial. Plasma (*n =* 6–11 animals per strain, each) and isolated glomeruli (*n =* 6–10 animals per strain, each) were prepared as previously described (Mangelsen et al., 2020). For kidney cortex preparation (*n =* 6–9 animals per strain, each) approximately 5–20 mg of tissue was accurately weighed and homogenized with 500 µL water. 50 μL aliquots were taken for protein measurement following Lowry protocol (Lowry et al., 1951) with primary alkaline hydrolysis (Mangelsen et al., 2020). For alkaline hydrolysis, 50 µL tissue suspensions were mixed with 16 µL 10 mol/L sodium hydroxide solution and incubated for 1 hour at 60°C. The pH values were adjusted to pH = 10 using 58% acetic acid. For prostaglandin measurement, the tissue suspensions were added with internal standard and BHT and further processed like plasma.

#### 2.5.2 LC/ESI-MS/MS

The residues were analyzed using the liquid chromatography tandem mass spectrometry protocol in an Agilent 1290 HPLC system with binary pump, multisampler and column thermostat with a Zorbax Eclipse plus C-18, 2.1 × 150 mm, 1.8 µm column using a solvent system of aqueous acetic acid (0.05%) and acetonitrile. The HPLC was coupled with an Agilent 6,495 Triplequad mass spectrometer (Agilent Technologies, Santa Clara, CA, United States) with electrospray ionisation source. Further details were given in Mangelsen et al. (2020) and Mangelsen et al. (2020). The analytes PGE_2_, 15-keto-PGE_2_, and 13,14-dihydro-15-keto-PGE_2_ were assessed. Glomerular and cortical values are normalized to protein, plasma values to ml of plasma and urinary values to creatinine. Analytes in isolated glomeruli and plasma in MWF rats and SHR at week 8, respectively, were recently published (Mangelsen et al., 2020).

#### 2.5.3 Metabolic ratios

For determination of metabolic ratios the quotient of PGE_2_/15-keto-PGE_2_ was calculated as a surrogate for 15-PGDH enzyme activity and the quotient of 15-keto-PGE_2_/13,14-dihydro-15-keto-PGE_2_ was determined as a surrogate for PTGRs enzyme activities. Low values represent higher enzymatic conversions and high values lower enzymatic conversions.

### 2.6 Statistics

Statistical analysis was performed using the SPSS Statistics 28.0.0.0. Data are presented as mean ± SD, and *p* < 0.05 was considered as statistically significant. Normal distribution was tested with the Shapiro-Wilk test. Data are normally distributed unless otherwise specified. Normally distributed data were compared by ANOVA. Results not normally distributed were analyzed by Kruskal–Wallis test and by Mann-Whitney *U* test as indicated. For identification of outliers, Grubbs’ outliers test (*α* = 0.05) was performed.

## 3 Results

### 3.1 Rat phenotypic characteristics

**FIGURE 2:**
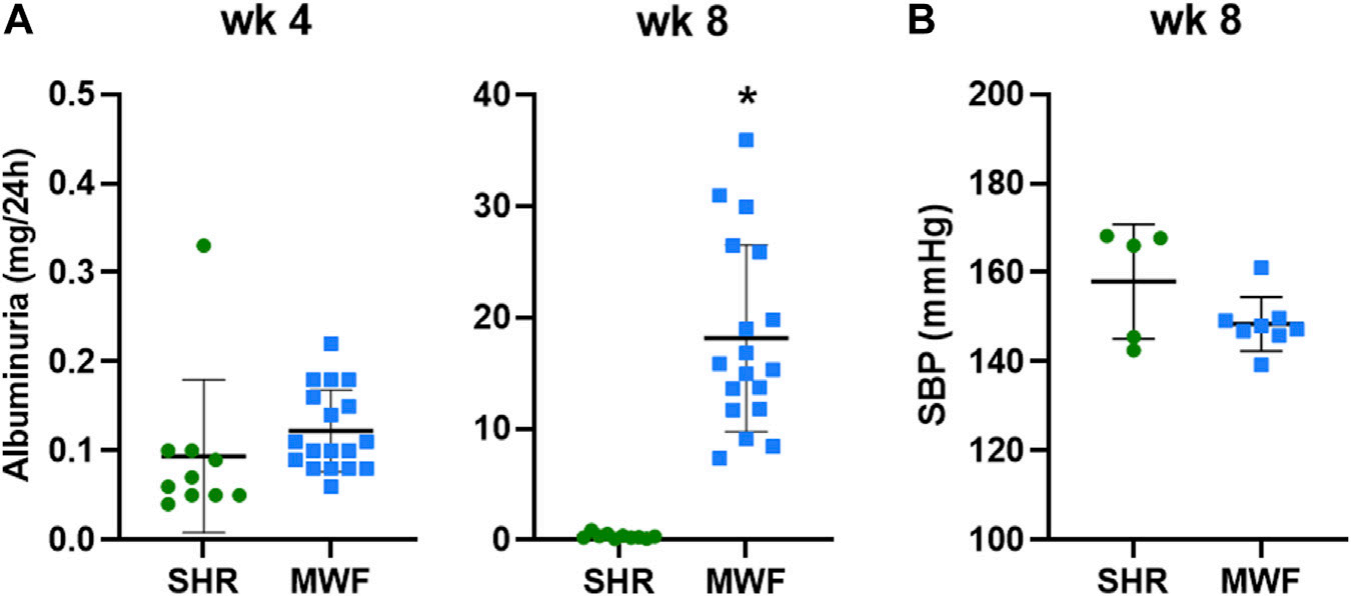
Phenotypic characteristics of Munich Wistar Frömter (MWF) and spontaneously hypertensive rats (SHR). **(A)** Urinary albumin excretion (UAE) at 4 and 8 weeks of age. MWF (*n* = 18), SHR (*n* = 10). **(B)** Systolic blood pressure (SBP) at 8 weeks of age. MWF (*n* = 8), SHR (*n* = 5). Analyte data were tested for normal distribution using Shapiro-Wilk test and analyzed by Mann-Whitney *U* test. Values shown as mean ± SD; *p < 0.0001 vs. SHR. UAE values were recently published (Schulz et al., 2019).

MWF rats and the contrasting albuminuria resistant SHR strain showed both similar UAE levels below 0.33 mg/24 h at 4 weeks of age (Figure 2A). In contrast, UAE at week 8 was significantly higher in MWF rats compared to the reference strain (*p* < 0.0001). Mean SBP values were, albeit numerically higher in SHR, not significantly different between MWF rats vs. SHR (148.4 ± 6.0 mmHg vs. 158.0 ± 12.8 mmHg) at 8 weeks of age (Figure 2B).

### 3.2 Lipidomic profile of PGE_2_ analytes and metabolic ratios

#### 3.2.1 PGE_2_ in kidney tissue, plasma and urine

**FIGURE 3:**
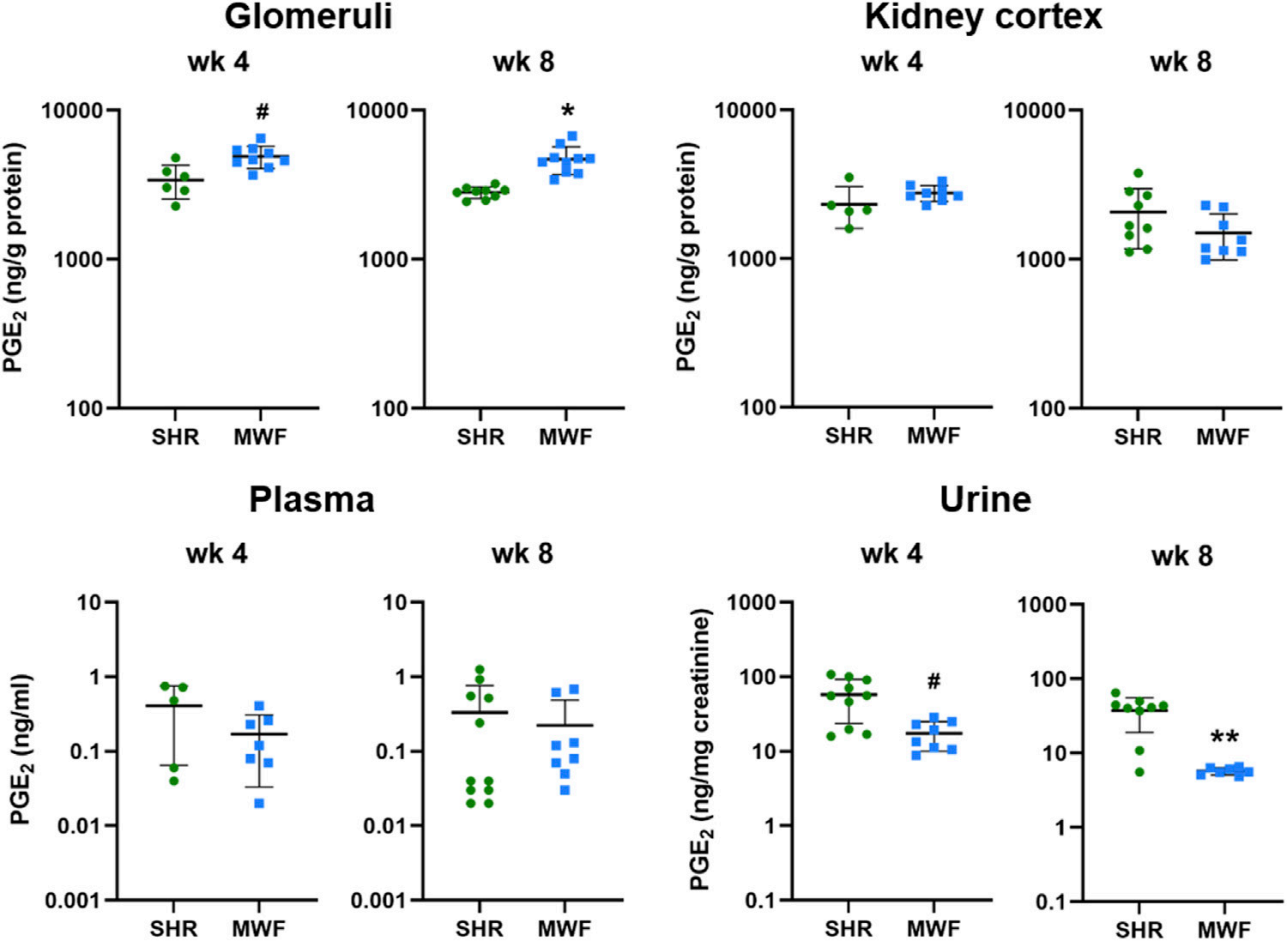
Metabolic levels of PGE_2_ in Munich Wistar Frömter (MWF) compared to spontaneously hypertensive rats (SHR). Analyte was assessed by LC/ESI-MS/MS in isolated glomeruli kidney cortex, plasma, and urine in 4 and 8 week old animals. *n* = 5–11 animals per rat strain. Data plotted as mean ± SD; logarithmic scale is used for visualization. *p < 0.0001, **p < 0.001, and #*p* < 0.02, respectively. Analyte data were tested for normal distribution using Shapiro-Wilk test. Normally distributed analytes were analyzed by one-way ANOVA, not normally distributed analytes by Kruskal–Wallis test and by Mann-Whitney *U* test.

Glomerular levels of PGE_2_ were increased in MWF at week 4 and 8 (*p* < 0.006, respectively), whereas no difference for cortical levels was observed (Figure 3). Plasma levels of PGE_2_ did not differ between strains, while urinary PGE_2_ was significantly lower in MWF rats compared to SHR at both time points (*p* < 0.005, respectively; Figure 3).

#### 3.2.2 15-keto-PGE_2_ and 13,14-dihydro-15-keto-PGE_2_ metabolites in kidney tissue, plasma and urine

**FIGURE 4:**
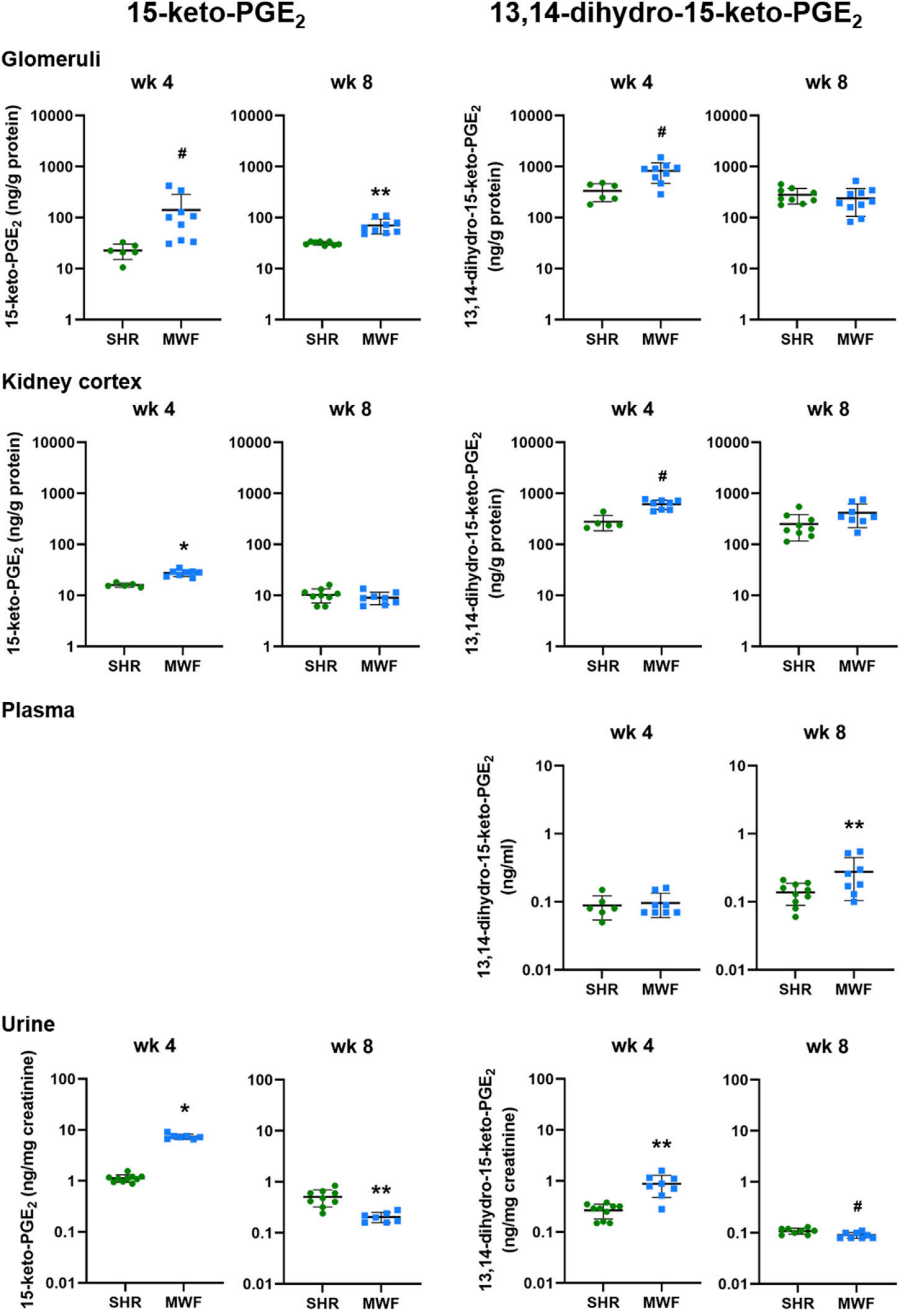
Metabolic levels of 15-keto-PGE_2_ and 13,14-dihydro-15-keto-PGE_2_ in Munich Wistar Frömter (MWF) rats compared to spontaneously hypertensive rats (SHR). Analytes were assessed by LC/ESI-MS/MS in isolated glomeruli kidney cortex, plasma, and urine in 4 and 8 week old animals. Plasma values for 15-keto-PGE_2_ were under the detection limit. *n* = 5–10 animals per rat strain. Data plotted as mean ± SD; logarithmic scale is used for visualization. *p < 0.0001, **p < 0.001, and #*p* < 0.02, respectively. Analyte data were tested for normal distribution using Shapiro-Wilk test. Normally distributed analytes were analyzed by one-way ANOVA, not normally distributed analytes by Kruskal–Wallis test and by Mann-Whitney *U* test.

Glomerular levels of 15-keto-PGE_2_ were significantly higher in MWF at week 4 and 8 (*p* < 0.006, respectively; Figure 4). In contrast, 13,14-dihydro-15-keto-PGE_2_ was only significantly higher at week 4 compared to SHR (*p* = 0.0071). In kidney cortex, 15-keto-PGE_2_ and 13,14-dihydro-15-keto-PGE_2_ were increased in MWF at week 4 (*p* < 0.0004, respectively), whereas no difference for cortical levels of PGE_2_ metabolites was observed at week 8 (Figure 4). Glomerular PGE_2_ levels were remarkably up to 150-fold higher than the respective 15-keto-PGE_2_ levels, whereas cortical PGE_2_ levels were even up to 200-fold higher than the corresponding 15-keto-PGE_2_ levels. Glomerular 13,14-dihydro-15-keto-PGE_2_ levels were elevated up to 15-fold compared to the 15-keto-PGE_2_ levels, whereas cortical levels were up to 46-fold higher than the corresponding 15-keto-PGE_2_ levels (Figures 3, 4).

Plasma levels of 13,14-dihydro-15-keto-PGE_2_ did not differ between strains at week 4 but significantly increased in MWF at week 8 (*p* < 0.03; Figure 4). 15-keto-PGE_2_ was below the detection limit in plasma.

Urinary 15-keto-PGE_2_ and 13,14-dihydro-15-keto-PGE_2_ levels were significantly increased in MWF rats compared to SHR at week 4 (*p <* 0.0005, respectively; Figure 4). Both analytes were significantly decreased in urine of MWF with increased albuminuria at week 8 (*p <* 0.02 vs. SHR, respectively; Figures 2, 4).

#### 3.2.3 Glomerular metabolic ratios of 15-PGDH and PTGRs

**FIGURE 5:**
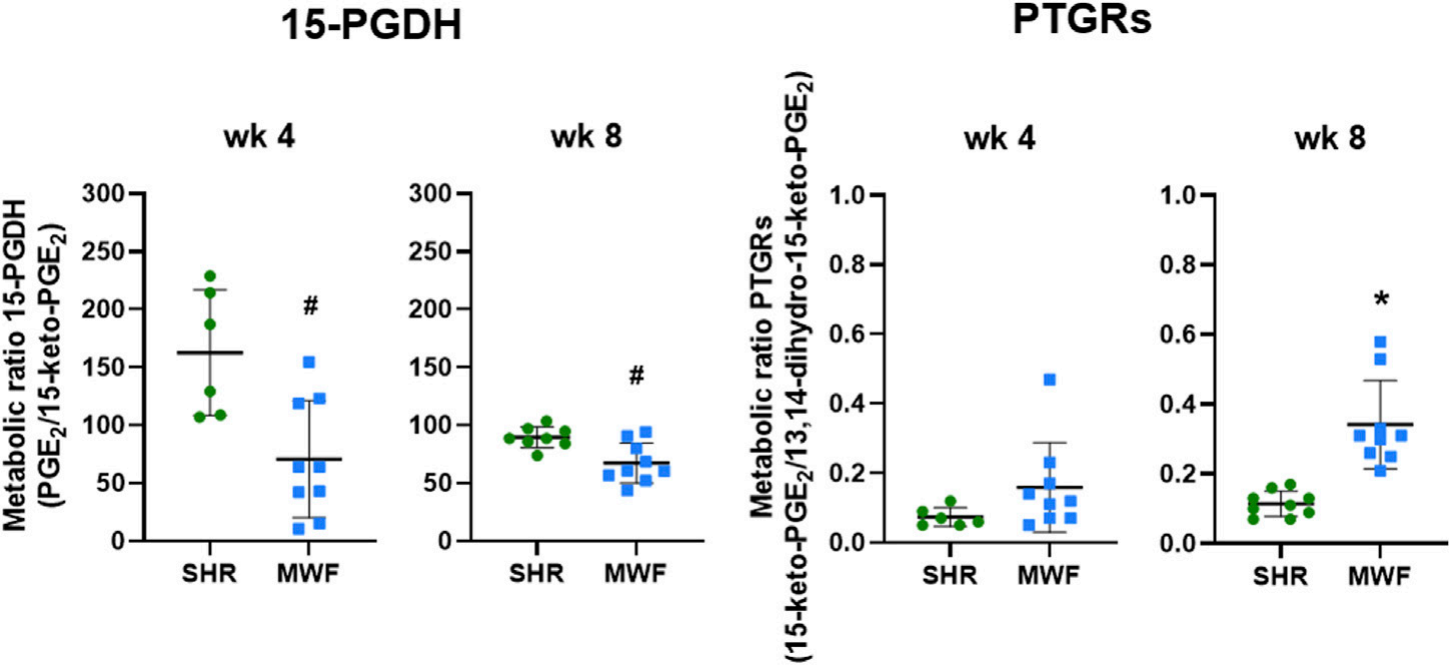
Metabolic ratios of 15-hydroxyprostaglandin dehydrogenase (15-PGDH) and prostaglandin reductases (PTGRs) in isolated glomeruli of Munich Wistar Frömter (MWF) rats compared to spontaneously hypertensive rats (SHR). Metabolic ratios of PGE_2_/15-keto-PGE_2_ were calculated as a surrogate for 15-PGDH activity and metabolic ratios of 15-keto-PGE_2_/13,14-dihydro-15-keto-PGE_2_ were assessed as a surrogate for PTGRs activities at 4 and 8 weeks of age. Values were plotted as mean ± SD. *p < 0.0001, #*p* < 0.04, respectively. Data were tested by one-way ANOVA.

Glomerular metabolic ratio of PGE_2_/15-keto-PGE_2_ as a surrogate for 15-PGDH enzyme activity showed significantly lower metabolic ratios in MWF compared to SHR at week 4 and week 8 (*p* < 0.006, respectively; Figure 5). Metabolic ratios of 15-keto-PGE_2_/13,14-dihydro-15-keto-PGE_2_ as a surrogate for PTGRs enzyme activities were similar between the strains at week 4, but significantly increased in MWF compared to SHR at week 8 (*p* < 0.0001, respectively; Figure 5).

### 3.3 Glomerular mRNA expression analysis of PGE_2_ degrading enzymes

**FIGURE 6:**
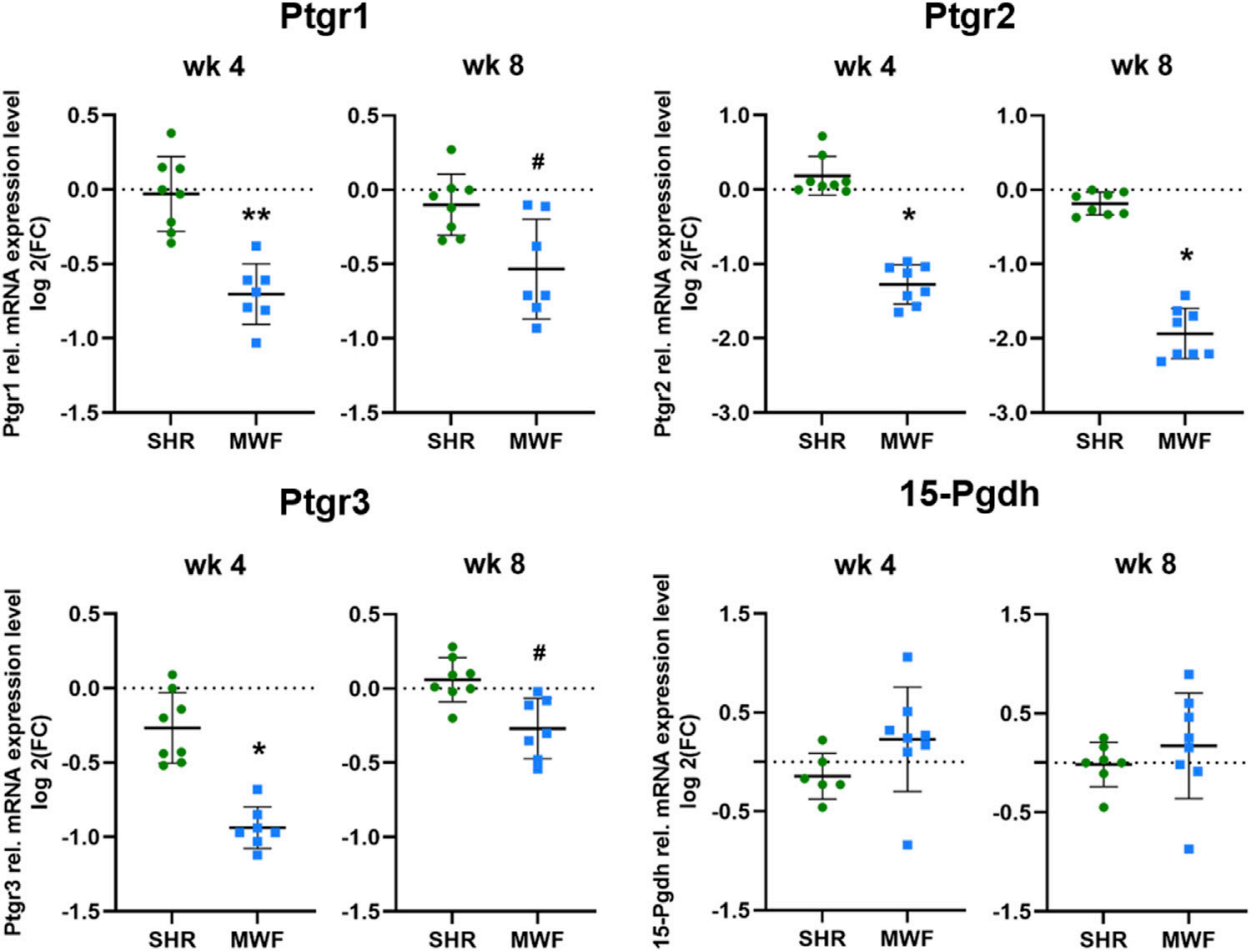
Targeted gene expression analyses of prostaglandin E2 (PGE_2_) degrading enzymes. Quantitative real-time PCR (qPCR) was performed for prostaglandin reductase (*Ptgr*)*1-3* and 15-hydroxyprostaglandin dehydrogenase (*15-Pgdh*) in isolated glomeruli tissue. *Ptgr1-3* showed significantly reduced expression values in Munich Wistar Frömter (MWF) compared to spontaneously hypertensive rats (SHR) at 4 and 8 weeks of age. *15-Pgdh* was similar between the strains. *n* = 6–8, each; values are plotted as mean ± SD. *p < 0.0001, **p < 0.001, and *#p* < 0.02, respectively. Gene data were tested for normal distribution using Shapiro-Wilk test: all genes were normally distributed and were analyzed by one-way ANOVA. Quantitative mRNA levels were normalized by the reference gene hydroxymethylbilane synthase (*Hmbs*, *Pbgd*).

mRNA expression of *Ptgr1, Ptgr2, and Ptgr3* demonstrated significantly lower expression levels in MWF rats as compared to SHR at both time points (*p* < 0.02, respectively; Figure 6). In contrast, *15-Pgdh* mRNA expression was similar between the strains.

## 4 Discussion

Within the different signaling mechanisms of PGs in the kidney, PGE_2_ increases GFB permeability (Nasrallah et al., 2014) and its upregulation in podocytes is linked to a response to increased FFSS as observed during GH (Srivastava et al., 2010; Srivastava et al., 2020). Moreover, PGE_2_ upregulation in podocytes also associates with actin cytoskeleton rearrangement (Martineau et al., 2004) and the latter contributes to podocyte foot process effacement (Kaplan et al., 2000) and slit diaphragm damage (Tryggvason and Wartiovaara, 2005), which is crucial for albuminuria development. A pathogenic role of signaling via the EP2 and possibly with lesser relevance via EP4 has been also suggested (Penn et al., 2001). We recently demonstrated in a yeast model that PGE_2_ and its downstream metabolite 15-keto-PGE_2_ bind to both EP2 and EP4 *in vitro* (Kourpa et al., 2022). Moreover, we showed in lipidomic analyses by LC/ESI-MS/MS that concerted EP2 and EP4 signaling mediates autocrine PGE_2_ signaling in human podocytes (Mangelsen et al., 2020). In a diabetic mouse model, expression of EP4 was detected in glomeruli and EP4 blockade could significantly ameliorated albuminuria development (Guan et al., 2022). Moreover, selective EP4 blockade is protective in a model of subtotal nephrectomy (Thieme et al., 2017). In contrast, inhibition of EP2 and activation of EP4 has the strongest effect in decreasing albuminuria in a hyperfiltration-induced injury mouse model with unilaterally nephrectomy (Srivastava et al., 2022). In the non-diabetic MWF rat model of CKD with GH, we showed a renoprotective effect of combined EP2/EP4 receptor inhibition of the COX2-PGE_2_-EP2/EP4 axis, since dual receptor blockade during onset of albuminuria development ameliorated albuminuria in this model, while systemic arterial blood pressure and GFR were not affected (Kourpa et al., 2023). Taken together, EP2 and EP4 receptors are potential targets for therapeutical intervention in hyperfiltration-induced glomerular injury.

15-keto-PGE_2_ and 13,14-dihydro-15-keto-PGE_2_ have been considered biologically inactive for a long time. Recently, a bioactive role of 15-keto-PGE_2_ has been identified and its signaling through activation of the peroxisome proliferator activated receptor gamma (PPAR-γ) pathway investigated (Chou et al., 2007; Lu et al., 2014; Chang et al., 2016; Chen et al., 2018). Effects of 15-keto-PGE_2_ are mediated via EP2 and EP4 receptors *in vitro* and *in vivo* (Endo et al., 2020; Kourpa et al., 2022). Previously, it has been shown that 15-keto-PGE_2_ affects the glomerular morphology of zebrafish embryonic kidney (Kourpa et al., 2023). However, the activity of PGE_2_ degrading enzymes and thus the metabolic pathway of COX2-PGE_2_ have not yet been implicated in renal physiology nor their potential contribution to the initiation and/or progression of CKD, i.e., albuminuria (Nasrallah et al., 2014; Srivastava et al., 2014; Chen et al., 2018). This indicates the need to analyze the metabolic downstream pathway of COX2-PGE_2_ in more detail. To this end we used the LC/ESI-MS/MS methodology for exact quantification in different kidney tissues and body fluids in the time window of albuminuria development in the MWF model system.

Our lipidomic profiling revealed elevated glomerular PGE_2_ levels, which were accompanied with reduced urinary PGE_2_ levels in MWF rats compared to SHR (Figure 3). In contrast, elevated urinary PGE_2_ levels were previously linked to glomerular injury and considered as a potential biomarker for early stages of adaptive hyperfiltration-induced injury preceding albuminuria in children (Srivastava et al., 2014; Srivastava et al., 2020). Notable, urinary 13-14-dihydro-15-keto-PGE_2_ levels were downregulated in an unilateral nephrectomized mouse model for GH with albuminuria reduction due to EP2 antagonist and EP4 agonist treatment (Srivastava et al., 2022). It should be however noted here, that the evaluation of urinary differences of PG levels are difficult to interpret. Hence, the origin of PGs in urine is unclear, since it is not possible to dissect which fractions of PGs are possibly generated in the tubular/tissue compartment and how much is attributable to glomerular filtration. However, to clarify this, further investigations including lipidomic analysis of PGE_2_ metabolites in tubular compartment could complement our findings.

Differences between glomerular and cortical PGE_2_ analytes highlight the importance of choosing the right compartment, i.e., glomerular tissue, when addressing glomerular questions. We detected elevated PGE_2_ levels in isolated glomeruli of MWF rats at both investigated time points compared to SHR. Cortical analyses did not show those significant changes between rat strains emphasizing the need to isolate glomeruli. We observed remarkably higher glomerular and cortical PGE_2_ levels than the respective 15-keto-PGE_2_ levels at both time points and elevated 13-14-dihydro-15-keto-PGE_2_ levels compared to the 15-keto-PGE_2_ levels, respectively. Hence, we conclude that the PTGRs are not the rate-limiting enzymes in the PGE_2_ degradation and inactivation in the kidney, rather 15-PGDH seems to be rate limiting in this process.

In contrast to the analysis of glomerular tissue, lipidomic analysis in plasma was not informative when comparing MWF rats and SHR during onset of albuminuria development. Comparing these results with previously reported PGE_2_ plasma levels in older MWF rats as measured by ELISA technique (Ulu et al., 2009), we measured almost four times lower average PGE_2_ plasma level. This discrepancy emphasizes the importance to use sensitive and precise state-of-the-art LC/ESI-MS/MS methodology to measure PGE_2_ lipids. Mass spectrometric analyses are preferable due to their higher specificity and selectivity compared to ELISA and standardized measurement procedures are lacking within different ELISA kits (Faupel-Badger et al., 2010; Gandhi et al., 2017). In conclusion, we observed a dysregulation of glomerular PGs but no dysregulation in plasma levels.

Metabolic ratios as a surrogate for enzyme activity revealed reduced glomerular 15-PGDH activity in MWF compared to SHR (Figure 5), while glomerular mRNA expression of *15-Pgdh* was similar between strains. The increased glomerular PGE_2_ values in MWF rats could be thus at least partially due to the lower 15-PGDH activity and a reduced PGE_2_ degradation.

In summary, our study highlights the importance of utilizing sensitive mass spectrometry technology for PG measurement in body liquids and tissues. Furthermore, we emphasized the importance of investigating the PGE_2_ pathway in kidney tissues and more precisely in glomeruli as analysis of plasma and urine samples have limiting relevance here. We demonstrated for the first-time age-dependent dynamic changes in the PGE_2_ metabolic pathway, which is involved in GH in the MWF rat model. Enzymatic functions of PGE_2_ degrading enzymes support a potential causative mechanism for kidney physiology and albuminuria onset in the setting of GH.

## Data Availability

All relevant data is contained within the article: The original contributions presented in the study are included in the article/supplementary material, further inquiries can be directed to the corresponding author.
