# Supplementary material for: Tissue lipidomic profiling supports a mechanistic role of the prostaglandin E2 pathway for albuminuria development in glomerular hyperfiltration
Source: Front Netw Physiol. 2023 Dec 22;3:1271042. doi: 10.3389/fnetp.2023.1271042 (PMC10777844; doi:10.3389/fnetp.2023.1271042)
Supplement: Supplementary file 1 [file DataSheet1.pdf]

Supplementary Material

Tissue lipidomic profiling supports a mechanistic role of the prostaglandin E2 pathway for albuminuria development in glomerular hyperfiltration

Debora Kaiser-Graf[1], Angela Schulz[1*], Eva Mangelsen[1], Michael Rothe[2], Juliane Bolbrinker[1], Reinhold Kreutz[1]

*** Correspondence:** Dr. Angela Schulz, Charité – Universitätsmedizin Berlin, corporate member of Freie Universität Berlin and Humboldt-Universität zu Berlin, Institute of Clinical Pharmacology and Toxicology, Charitéplatz 1, 10117 Berlin, Germany
angela-martina.schulz@charite.de

1 Supplementary Figures and Tables

1.1 Supplementary Figures

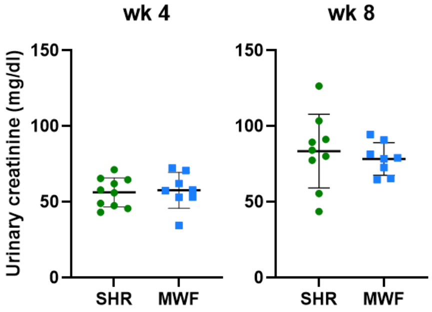

Supplementary Figure 1. Urinary creatinine of Munich Wistar Frömter (MWF) and spontaneously hypertensive rats (SHR) at 4 and 8 weeks of age determined by Jaffé method, MWF (*n* = 8-9), SHR (*n* = 9-11). Values shown as mean ± SD. Analytes data were analyzed by one-way ANOVA.

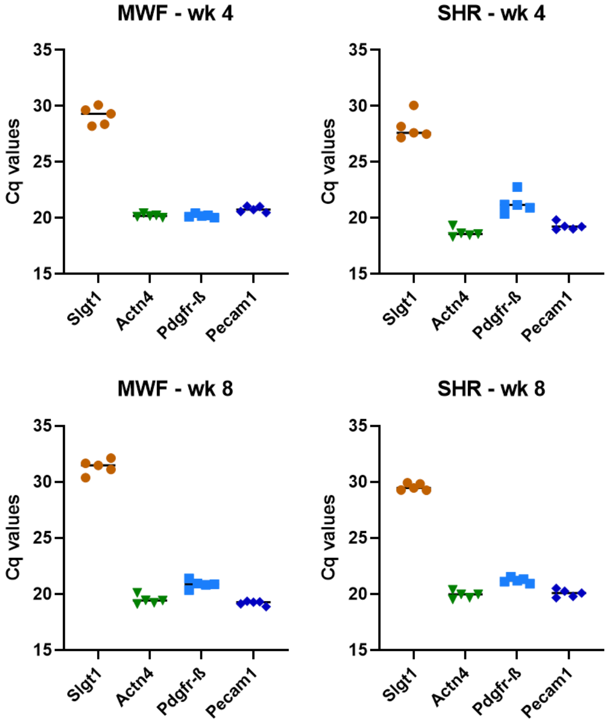

Supplementary Figure 2. Assessment of glomerular purity by genetic marker analyses in isolated glomeruli by qPCR. Cq values of the respective marker gene to the reference gene *Pbgd* were shown for solute carrier family 5 member 1 (*Slc5a1*, *Sglt1*), alpha actinin 4 (*Actn4*), platelet derived growth factor receptor beta (*Pdgfrb*), and platelet endothelial cell adhesion molecule-1 (*Pecam1*, *CD31*) in Munich Wistar Frömter (MWF) and spontaneously hypertensive rats (SHR) at 4 and 8 weeks of age

(*n* = 5, each). Evaluation of Cq values of glomerular markers showed abundant target nucleic acid in the samples (Cq values between 18 and 23). A moderate amount of nucleic acids of *Slc5a1* was detected in all samples (Cq values between 27 and 33), resulting in small contamination by tubular fractions in our isolated glomeruli as expected.
